# Atmosphere particulate matter and respiratory diseases during COVID-19 in Korea

**DOI:** 10.1038/s41598-024-59643-x

**Published:** 2024-05-02

**Authors:** Ji Young Hong, Taemo Bang, Sun Bean Kim, Minwoo Hong, Jaehun Jung

**Affiliations:** 1 Division of Pulmonary, Allergy and Critical Care Medicine, Department of Internal Medicine, Chuncheon Sacred Heart Hospital, Hallym University Medical Center, Chuncheon-si, Gangwon-do 24253 Republic of Korea; 2 AI Product Team, Gmarket, Seoul, Republic of Korea; 3 Department of Internal Medicine, Division of Infectious Diseases, Korea University College of Medicine, Seoul, Republic of Korea; 4 Department of Preventive Medicine, Gachon University College of Medicine, 38-13, Dokjeom-ro 3beon-gil, Namdong-gu, Incheon, 21565 Republic of Korea; 5 Artificial Intelligence and Big-Data Convergence Center, Gil Medical Center, Gachon University College of Medicine, Incheon, Republic of Korea

**Keywords:** Infectious diseases, Respiratory tract diseases, Risk factors

## Abstract

We aimed to examine the impact of COVID-19 non-pharmaceutical interventions (NPIs) on the relationship between air pollutants and hospital admissions for respiratory and non-respiratory diseases in six metropolitan cities in South Korea. This study compared the associations between particulate matter (PM_10_ and PM_2.5_) and hospital admission for respiratory and non-respiratory diseases before (2016–2019) and during (2020) the implementation of COVID-19 NPIs by using distributed lag non-linear models. In the Pre-COVID-19 period, the association between PM_10_ and admission risk for asthma and COPD showed an inverted U-shaped pattern. For PM_2.5_, S-shaped and inverted U-shaped changes were observed in asthma and COPD, respectively. Extremely high and low levels of PM_10_ and extremely low levels of PM_2.5_ significantly decreased the risk of admission for asthma and COPD. In the Post-COVID-19 outbreak period, the overall cumulative relationship between PM_10_ and PM_2.5_ and respiratory diseases and the effects of extreme levels of PM_10_ and PM_2.5_ on respiratory diseases were completely changed. For non-respiratory diseases, PM_10_ and PM_2.5_ were statistically insignificant for admission risk during both periods. Our study may provide evidence that implementing NPIs and reducing PM_10_ and PM_2.5_ exposure during the COVID-19 pandemic has contributed to reducing hospital admissions for environment-based respiratory diseases.

## Introduction

The COVID-19 pandemic has caused major changes in public health measures^1^. Governments worldwide responded by implementing a spectrum of control measures, ranging from stay-at-home orders to social distancing protocols and, in some instances, strict lockdowns, all aimed at curbing the transmission of the virus. Concurrently, non-pharmaceutical interventions (NPIs), such as hand hygiene and droplet precautions, were rigorously enforced^2^.

The World Health Organization recognizes air pollution as a global environmental threat to human health^3^. Numerous studies have demonstrated the association between air pollution and cardiovascular diseases, including heart attacks, strokes, and irregular heart rhythms^4^. Furthermore, ambient air pollution has been identified as a significant causative and exacerbating factor in various respiratory conditions, such as chronic obstructive pulmonary disease (COPD), asthma, and lung cancer^5,6^. Notably, more than 25% of premature deaths associated with air pollution are estimated to be respiratory in nature^7^.

The COVID-19 pandemic prompted unprecedented restrictions on travel and the suspension of industrial activities, resulting in a discernible improvement in air quality^8^. Similarly, in Korea, the implementation of COVID-19 control measures demonstrated positive effects on air quality, subsequently preventing premature deaths and mitigating healthcare costs attributable to air pollution^9^.

The decrease in hospital admissions for respiratory diseases during the COVID-19 pandemic has been reported to be an effect of NPIs^10^. Within the spectrum of NPIs, filtering facemasks emerged not only as an effective measure in impeding the spread of novel respiratory viruses but also as a deterrent against the transmission of particulate matters (PMs)^11,12^.

This study posits the hypothesis that NPIs during the COVID-19 pandemic may have altered the effects of air pollutants on several diseases. Specifically, we conjecture that the relationship between air pollution factors and diseases may differ between the Pre-COVID-19 period (2016–2019) and the Post-COVID-19 outbreak period (2020). To scrutinize this hypothesis, we analyzed the incidence of hospital admission of several diseases according to PM_10_ and PM_2.5_ levels from 2016 to 2020.

## Methods

### Study design

The analysis encompassed six metropolitan cities in South Korea, namely Seoul, Incheon, Gwangju, Daejeon, Daegu, and Busan. Notably, in the case of Incheon, Ongjin-gun, and Ganghwa-gun were excluded due to geographical requirements. This city-centric approach, rather than a nationwide scope, was adopted to account for variations in air pollution sources and demographics associated with distinct geographical features of each city. The study employed a multivariate meta-analysis to consolidate results obtained from the analysis of each of the six major cities.

### Data collection

Data for this study were derived from three databases. Hospital admission information for respiratory diseases (asthma, COPD, pneumonia, influenza) and non-respiratory (cancer, diabetic ketoacidosis, hyperosmolar hyperglycemic state (DKA/HHS), intracranial haemorrhage (ICH), myocardial infarction (MI)) diseases from 2016 to 2020, categorized by metropolitan area, was sourced from the National Health Insurance Service (NHIS) customized research database^13^. Table 1 presents the ICD-10 codes corresponding to the inclusion criteria for each disease. Air pollution source data, daily concentrations of PM_10_ and PM_2.5_ (μg/m^3^), were obtained from AirKorea^14^.

**Table 1:** ICD-10 code for inclusion criteria.

ICD-10 code	Disease	Total cases
J45	Asthma	86,141
C00-C09, C10-C43, C45-C97	Cancer	250,471
J43-44, J46-47	Chronic obstructive pulmonary disease	48,714
E100-101, E110-111, E120, E130-131, E140-141	Diabetic ketoacidosis or hyperosmolar hyperglycemic state	5562
J09-11	Influenza	101,072
I60-62	Intracranial haemorrhage	25,640
I21	Myocardial infarction	23,804
J12-18	Pneumonia	250,069

Meteorological factor data were collected as covariates, utilizing daily information for each province from the Korea Meteorological Administration (KMA)^15^. The three databases were merged based on date and city, forming the dataset for analysis.

### Statistical analysis

We performed a statistical analysis in two stages. First, distributed lag non-linear models (DLNMs) were fitted to the six metropolitan cities in South Korea to evaluate the non-linear relationship between hospital admissions and air pollution. In the second stage, the estimated coefficients and variance–covariance matrices from DLNMs were used for a multivariate meta-analysis.

#### Distributed lag non-linear models

We used DLNMs to evaluate the health effects of air pollution:

$${\text{log}}\left(E\left({Y}_{ijt}\right)\right)={\mathrm{\alpha }}_{ij}+{\text{NS}}\left({{\varvec{A}}}_{ijt},{\text{df}}_{p}, {\text{lag}},{\text{df}}_{l}{;{\varvec{\beta}}}_{ij}\right)+{{\varvec{\gamma}}}_{ij}{{\varvec{M}}}_{\text{ijt}}+{{{\varvec{\delta}}}_{ij}{\varvec{S}}}_{\text{t}},$$

where ${Y}_{ijt}$ represents daily hospital admission cases on day t from the year 2016 to 2020, assuming a quasi-Poisson distribution with $E\left({Y}_{ijt}\right)={\upmu }_{t}$, $V\left({Y}_{ijt}\right)=\upphi {\mu }_{t}$; $i$ and $j$ denote a disease ($i=1, 2, \cdots , 8$; Asthma, COPD, Pneumonia, Influenza, Cancer, DKA or HHS, ICH, MI) and cities ($j=1, 2, \cdots , 6$; Seoul, Incheon, Gwangju, Daejeon, Daegu, Busan) respectively; ${\alpha }_{ij}$ is an intercept; ${{\varvec{A}}}_{ijt}$ is a *cross-basis* matrix for each air pollution to model bi-dimensional space describing simultaneously the relationship along the single pollutant and its distributed lag effects, where NS is a natural cubic spline determined by regression coefficients vector ${{\varvec{\beta}}}_{ij}$ to explain non-linear relationships between ${Y}_{ijt}$ and air pollution; ${\text{df}}_{p}$ and ${\text{df}}_{l}$ are degrees of freedom in the predictor space and degrees of freedom in the additional lag dimension, respectively; ${{\varvec{M}}}_{\text{ijt}}$ is a selected vector of meteorological factors as covariates, with linear effects defined by a regression coefficients vector ${{\varvec{\gamma}}}_{ij}$; ${{\varvec{S}}}_{\text{t}}$ is a Fourier vector modeling daily seasonality, with linear effects defined by a regression coefficients vector ${{\varvec{\delta}}}_{ij}$. We used only the first six Fourier terms for daily seasonality ($m=365$)^16^.

Challenges in fitting DLNMs include choosing a large number of hyperparameters and deciding which factors to include in the model as covariates^17^. However, there is still no well-known unified optimization algorithm. To address this, we proposed an optimization algorithm for single pollutant DLNM in Table 2, based on best subset selection, one of the traditional variable selection methods in linear regression analysis. First, we considered five meteorological factors as covariates: average temperature, average relative humidity, average wind speed, diurnal temperature range (DTR), and precipitation. Three hyperparameters were explored: 7 to 31 maximum lag days, and 2 to 5 degrees of freedom in both the predictor space (${\text{df}}_{p}$) and the additional lag dimension (${\text{df}}_{l}$). When optimizing the models, we used grid search and parallel processing. The optimization algorithm was applied to all models for each city, with an initial optimization in Seoul. Hyperparameters for the models in the remaining cities were set to match those in Seoul for subsequent multivariate meta-analysis.

**Table 2:** Optimization algorithm for single pollutant DLNM.

Step	Description
1	${\text{Y}}$ is a daily count data that originates from quasi-Poisson distribution. Depending on the type of outcome, it can be assumed as one of the exponential families of distributions
2	Consider ${2}^{k}$ of DLNMs by best subset selection, where $k$ is the number of covariates (e.g. meteorological factors) except the terms to describe seasonality, trend, holiday effects, etc. In other words, comparing the model performance by considering everything from the non-covariate single-exposure model to the full-covariates single-exposure model
3	Tune hyper-parameters of each ${2}^{k}$ of DLNM based on QAIC (the AIC for quasi-Poisson) • Maximum lag days: [${m}_{1},{m}_{2}, \cdots$] • Degrees of freedom in predictor space(${\text{df}}_{p}$): [${v}_{1},{v}_{2}, \cdots$] • Degrees of freedom in additional lag dimension(${\text{df}}_{l}$): [${l}_{1},{l}_{2}, \cdots$] Knots are equally spaced, and a natural cubic spline is selected as a basis function. To tune and optimize each ${2}^{k}$ of DLNM, use one of the search methods (e.g. grid search, random search)
4	Among the ${2}^{k}$ of DLNMs optimized in step 3, The model with the smallest QAIC value is selected as the best model. However, if there is a model with a QAIC difference of less than 2 from the optimal model with the smallest QAIC as follows: ${\Delta }_{{\text{i}}}={{\text{QAIC}}}_{{\text{i}}} -{{\text{QAIC}}}_{{\text{min}}}<2$ The simplest model is the best model by comparing the models, including the optimal one

*DLNM* distributed lag non-linear models.

Results of the analysis were summarized through visualizations based on the relative risk (RR), utilizing the median concentration of air pollutants in Seoul as the reference value. The software used for this analysis was R 4.1.3^18^, with the {dlnm} package for DLNM fitting^19^, and {foreach}^20^, {parallel}, {doParallel}^21^, {furrr}^22^ packages for algorithm implementation. Visualization was achieved using {ggplot2}^23^ and {patchwork}^24^.

#### Multivariate meta-analysis

Following the optimal DLNM fitting for each city, a fixed-effect multivariate meta-analysis was conducted. This involved combining results using estimated variance–covariance matrices and regression coefficients.

### Reproducible tutorial

To facilitate the application of the proposed optimization algorithm, a reproducible tutorial has been made available on GitHub^25^.

### Ethics approval and consent to participate

This study was approved by the Institutional Review Board of the Gachon University Gil Medical Center, Incheon, South Korea (IRB No. GCIRB2021-149), and participants informed consent was waived by the ethics committee of Gachon University Gil Medical Center because the data involved routinely collected medical data that was processed anonymously at all stages. All study methods were carried out based on the Declaration of Helsinki.

### AI statement

Generative AI was used for grammar and spelling, with no other applications.

## Results

### Summary statistics for patients and air pollutants

**Figure 1:**
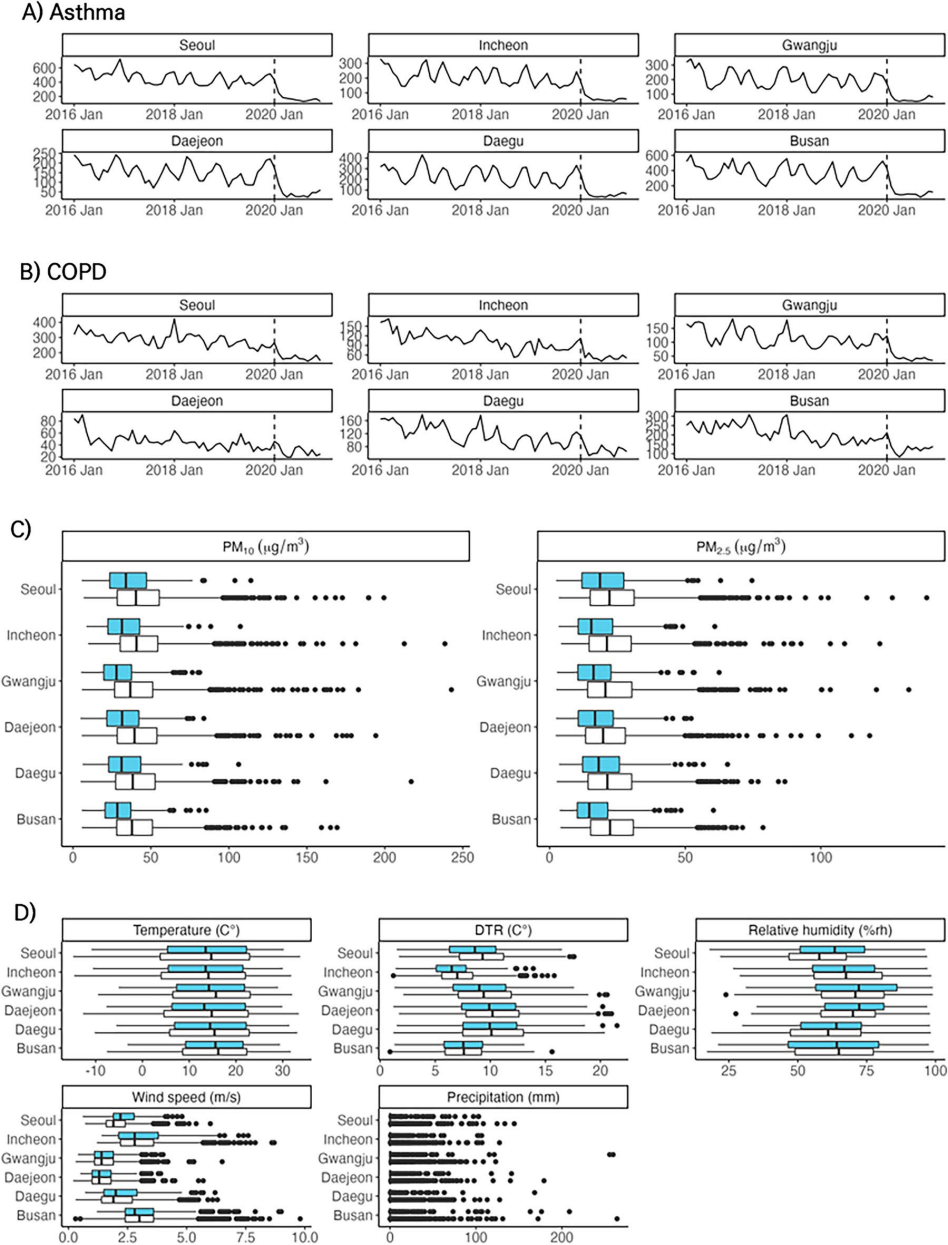
Monthly asthma and chronic obstructive pulmonary disease (COPD) admissions and box plots of meteorological factors. The monthly (**A**) Asthma and (**B**) COPD admissions in metropolitan cities in South Korea from 2016 to 2020. The area following the dashed line indicates the Post-COVID-19 outbreak period. (**C**) Box plots of air pollution by metropolitan cities, South Korea, from 2016 to 2020. Box plots filled with white and sky-blue represent the Pre-COVID-19 period (2016–2019) and Post-COVID-19 outbreak period (2020), respectively. (**D**) Box plots of meteorological factors by metropolitan cities in South Korea from 2016 to 2020, where the diurnal temperature range (DTR) is defined as the difference between daily maximum and minimum temperature. Boxplots filled with white and sky-blue represent the Pre-COVID-19 period (2016–2019) and Post-COVID-19 outbreak period (2020), respectively.

Table 1 provides the total number of admissions utilized in this analysis from 2016 to 2020. Substantial decreases in hospitalizations due to asthma and COPD were observed in the Pre-COVID-19 compared to Post-COVID-19 outbreak period (Fig. 1A and B). Similarly, hospital admissions for pneumonia and influenza exhibited significant reductions, while those for other non-respiratory diseases such as cancer, DKA/HHS, ICH, and MI remained relatively consistent (Supplementary Fig. 1).

As shown in Fig. 1C, the average PM_10_ and PM_2.5_ in the six metropolitan cities decreased during the intervention period. In contrast, meteorological factors, including average temperature, DTR, humidity, wind speed, and precipitation, did not show a significant difference between the Pre-COVID-19 and Post-COVID-19 outbreak periods **(**Fig. 1D).

### Distributed lag non-linear models

**Figure 2:**
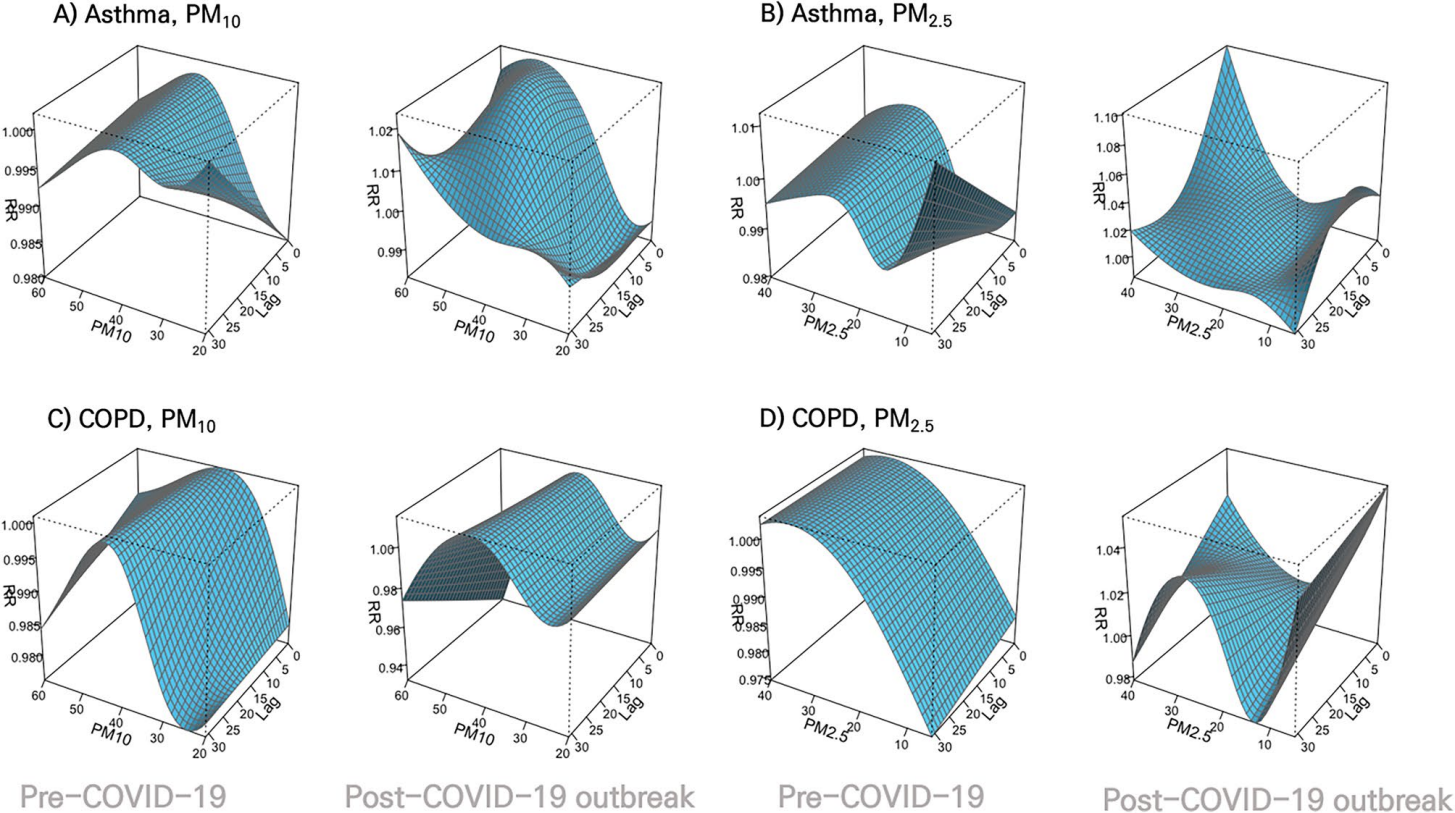
Overall ${\text{PM}}_{10}$ and ${\text{PM}}_{2.5}$ effect on admissions of Asthma and Chronic obstructive pulmonary disease (COPD). Overall ${\text{PM}}_{10}$ and ${\text{PM}}_{2.5}$ effect on admissions of Asthma and COPD by 31 lag days in the Pre-COVID-19 and Post-COVID-19 outbreak period as 3D plots for multivariate meta-analyses. (**A**) and (**B**) represent the results for Asthma. (**C**) and (**D**) represent the results for COPD.

We used the overall picture to visualize the effects of air pollutant variables on different lag days. The 3D plot of RR showed that the lag structures of air pollutants and respiratory diseases differed between the Pre-COVID-19 and Post-COVID-19 outbreak periods (Fig. 2, Supplementary Fig. 2).

**Figure 3:**
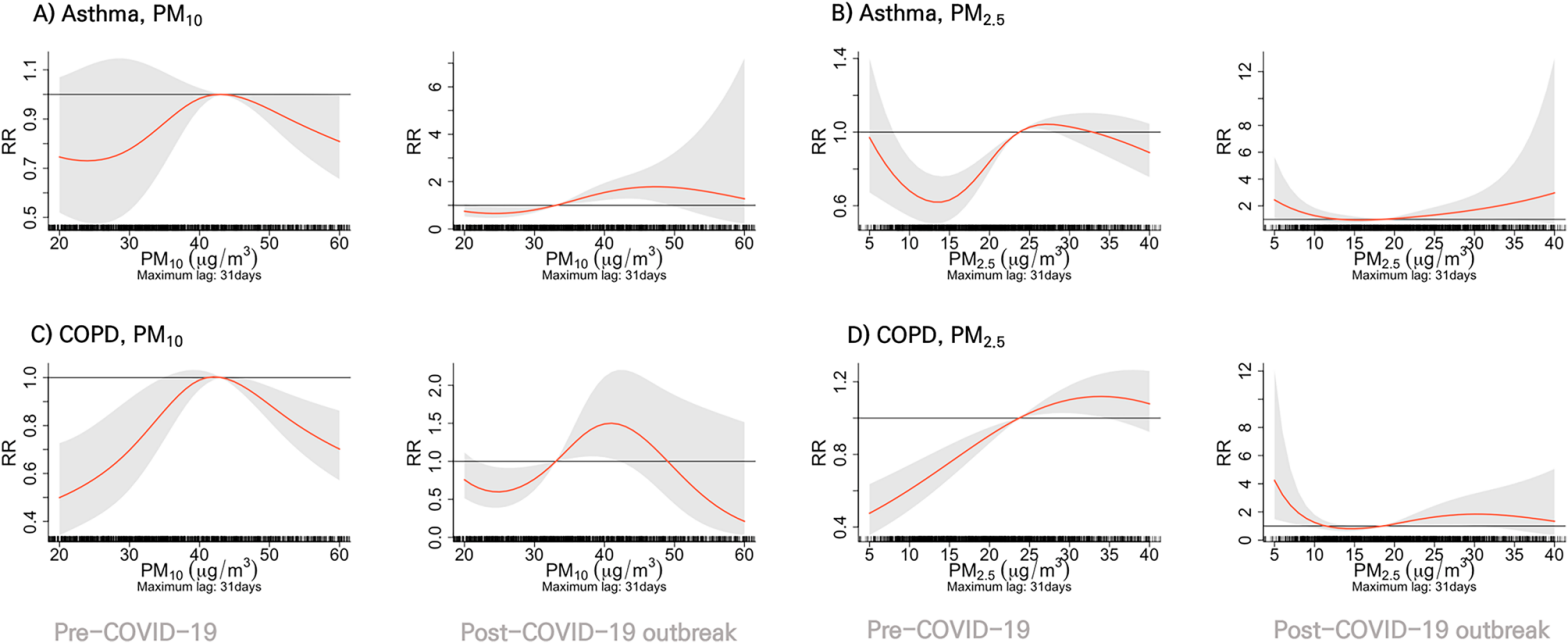
Cumulative ${\text{PM}}_{10}$ and ${\text{PM}}_{2.5}$ effect on admissions of Asthma and Chronic obstructive pulmonary disease (COPD). Cumulative ${\text{PM}}_{10}$ and ${\text{PM}}_{2.5}$ effect of 31 lag days on admissions of Asthma and COPD as overall cumulative association plots for multivariate meta-analyses. (**A**) and (**B**) represent the results for Asthma, which are shown in a row in the Pre-COVID-19 and Post-COVID-19 outbreak period. (**C**) and (**D**) represent the results for COPD, which are shown in a row in the Pre-COVID-19 and Post-COVID-19 outbreak period.

The estimated associations represented on the RR scale are illustrated in Fig. 3 to investigate the exposure–response analysis for admission rates of asthma and COPD with PM_10_ and PM_2.5_ before and after the COVID-19 pandemic over the entire lag period of 0–31 days. In the Pre-COVID-19 period, PM_10_ demonstrated an inverted U pattern for both asthma and COPD (Fig. 3A,C). The RR of admission exhibited a decline on either side of the peak PM_10_ level, approximately 40 μg/m^3^, with negative values. Conversely, during the Post-COVID-19 outbreak period, both diseases displayed a positive RR at PM_10_ concentrations ranging from 30–45 μg/m^3^ and 35–43 μg/m^3^ for asthma and COPD, respectively (Fig. 3A,C).

Similar to PM_10_, PM_2.5_ also showed distinctive effect on hospitalization for respiratory diseases before and after the COVID-19 pandemic. In asthma-related hospital admissions, a gradual S-shaped curve between 13 and 26 μg/m^3^ characterized the Pre-COVID-19 period, with negative RR values observed in the range of 8–23 μg/m^3^. Conversely, in the Post-COVID-19 outbreak period, the association of PM_2.5_ showed a U pattern for asthma and was statistically insignificant. For COPD in the Pre-COVID-19 period, the association of PM_2.5_ showed an inverted U pattern, with a negative RR below 23 μg/m^3^. In Post-COVID-19 outbreak period, a gradual S-shape curve between 14 and 30 μg/m^3^ was observed, with a positive RR below 8 μg/m^3^ (Fig. 3B,D).

**Figure 4:**
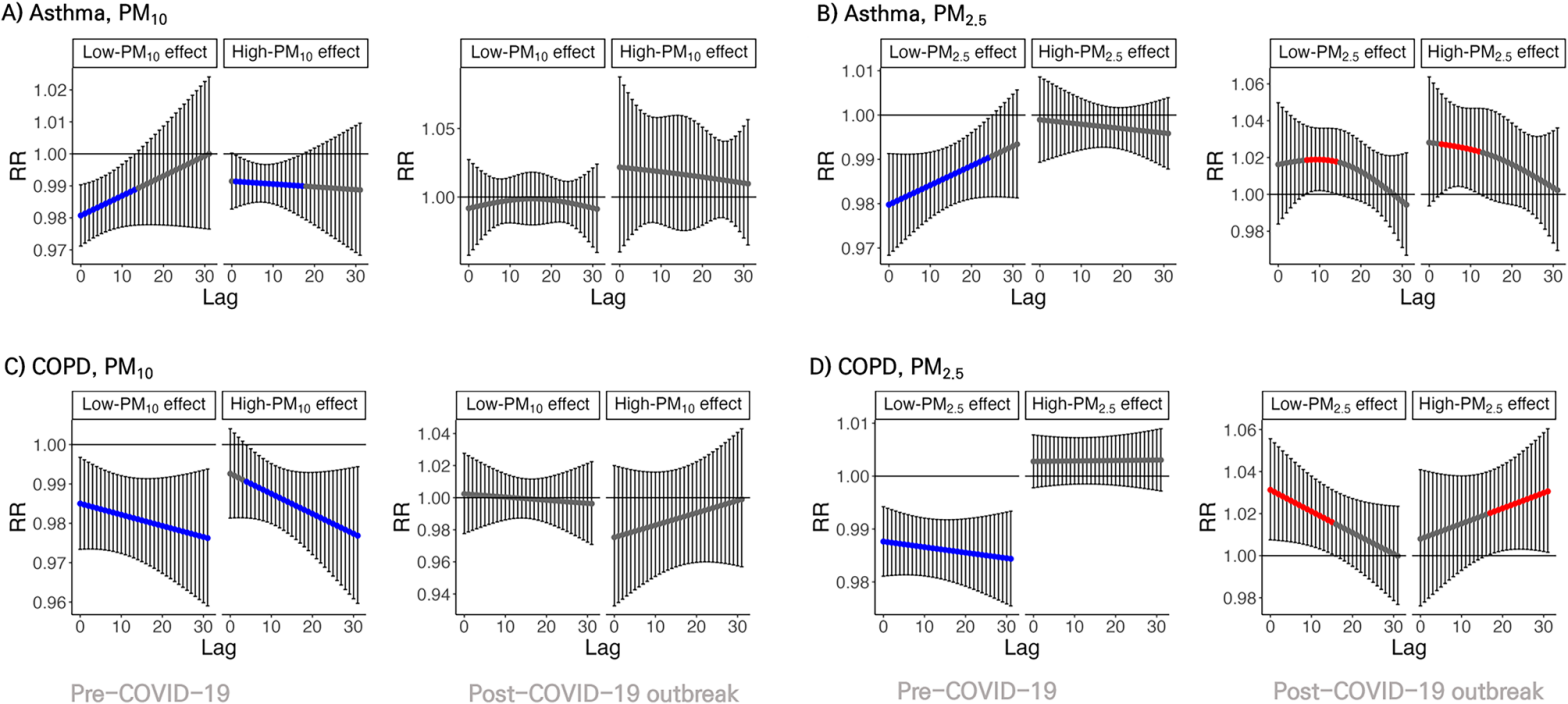
Extreme effect of ${\text{PM}}_{10}$ and ${\text{PM}}_{2.5}$ on admissions of Asthma and Chronic obstructive pulmonary disease (COPD). Extreme effect of ${\text{PM}}_{10}$ and ${\text{PM}}_{2.5}$ on admissions of Asthma and COPD as high-low effect plots for multivariate meta-analyses. High effect and low effect mean a 90th quantile value versus the median value of $\mathrm{each }\;{\text{PM}}$ in Seoul and a 10th quantile value versus the median value of $\mathrm{each }\;{\text{PM}}$ in Seoul, respectively. The dot means the point estimator of the relative risk, and the bar means the 95% interval. If the confidence interval is greater than 1, the dot has a red colour, and if it is less than 1, it has a blue colour. (**A**) and (**B**) represent the results for Asthma, which are shown in a row in the Pre-COVID-19 and Post-COVID-19 outbreak period. (**C**) and (**D**) represent the results for COPD, which are shown in a row in the Pre-COVID-19 and Post-COVID-19 outbreak period.

Figure 4 shows the effect of extremely high and low levels of PM_10_ and PM_2.5_ on admissions for asthma and COPD at different lag times, up to 31 days. In the Pre-COVID-19 period, the protective effect of both high and low PM_10_ on hospitalization due to asthma was significant for up to 14 days, While for COPD, effects gradually increased with longer lag days. However, in the Post-COVID-19 outbreak period, lag effects for high and low PM_10_ were not significant. Extremely low PM_2.5_ significantly decreased the risk of admission for both asthma and COPD during the Pre-COVID-19 period. The protective effect of PM_2.5_ was significant until a minimal lag of 20 days in both diseases and increased over time in COPD. In contrast, in the Post-COVID-19 outbreak period, extremely low and high PM_2.5_ increased the risk of asthma and COPD. The deteriorated effect on COPD at extremely low levels was most pronounced at lag 0 days, and reduced at later lag times, while the effect increased over time at extremely high levels.

For pneumonia, the association of PM_2.5_ showed an inverted U pattern with a negative RR below 23 μg/m^3^ in the Pre-COVID-19 period but a U pattern in the Post-COVID-19 outbreak period (Supplementary Fig. 3B). The protective effect of PM_2.5_ on pneumonia was most prominent at lag 0 days (Supplementary Fig. 4B).

As for influenza, in the Pre-COVID-19 period, the RR was highest at low extreme levels for PM_10_ and PM_2.5,_ decreasing as concentrations increased (Supplementary Fig. 3C,D). Moreover, the effect of low and high PM_10_ and PM_2.5_ on influenza was the largest at lag 0 days and statistically significant up to more than 20 days (Supplementary Fig. 4C,D). However, in the Post − COVID-19 outbreak period, the confidence intervals (CI) of RR at all values were wide and statistically insignificant (Supplementary Fig. 3C,D).

In non-respiratory diseases, for most parts of PM_10_ and PM_2.5_ concentration ranges, the values were statistically insignificant, with the 95% CI overlapping the RR of 1 both before and after the COVID-19 pandemic. An exception was noted for PM_10_ concentrations ranging from 27 to 40 μg/m^3^, showing an incremental effect on the RR of admission for cancer (Supplementary Fig. 3). In addition, lag effects for both periods were insignificant across all non-respiratory diseases (Supplementary Fig. 4).

## Discussion

During the COVID-19 epidemic, numerous countries reported a substantial decrease in admissions for respiratory diseases, including COPD and asthma^10,26,27^. In this study, we sought to interpret this phenomenon from the perspective of air pollution, applying DLNMs to explore the relationship between air pollution and hospital admissions for several diseases before and after the COVID-19 pandemic.

The effects of air pollutants on various diseases have been widely reported^4,28^. Prior studies have reported associations with exacerbation of respiratory diseases, emergency department visits, and hospital admissions^29^. PM induces inflammation and lung damage through mechanisms such as impairing antimicrobial activity and mucociliary transport^30,31^. Additionally, PM induces lung injury by producing reactive oxygen species, leading to oxidative stress and tissue damage^32^. Consistent with previous studies^33–35^, our data revealed a relationship between hospitalization rates for respiratory diseases and PM_10_ and PM_2.5_ in the Pre-COVID-19 period. COPD exacerbation cases from the Korean nationwide database showed an inverted U-shaped pattern for PM_10_ and PM_2.5_^33^. Similarly, a study from China evaluating the association between asthma hospitalizations and PM_10_ and PM_2.5_ showed a non-linear pattern similar to ours^34^. Huh et al. reported the incidence of pneumonia increased up to approximately 20 μg/m^3^ of PM_2.5,_ showing an inverted U relationship^35^.

Conflicting results exist in the literature regarding influenza^36,37^. While Toczylowski reported an exponential relationship between cumulative PM_2.5_ pollution and the incidence of influenza-like illnesses (ILI)^37^, our results, akin to Wang et al.^38^, indicated that slightly low concentrations of PM2.5 were more associated with contaminant-related influenza. This may be attributed to behavioral factors and heightened healthcare awareness during poor air quality, leading people to adopt protective measures, including face masks, potentially mitigating the association between influenza hospitalization and PM concentration. Air pollutants, especially PM_10_ and PM_2.5,_ increase the incidence of ILI and induce greater healthcare utilization for acute lower respiratory infections^36^. Airborne pollution particles provide condensation nuclei for virus-droplet attachment^39^. As face masks are being worn at all times during the COVID-19 pandemic, the association between influenza hospitalization and PM concentration has disappeared.

Importantly, our results unveiled a considerable shift, as depicted in Figs. 3 and 4, in the relationship between hospitalization rates for respiratory diseases and PM_10_ as well as PM_2.5_ during the COVID-19 pandemic compared to Pre-COVID-19 period. Two unique phenomena related to the COVID-19 pandemic could explain these findings. Firstly, mitigation measures, such as travel restrictions and discontinuation of nonessential social gatherings, likely reduced exposure to ambient environmental triggers, including pollutants and PMs^40^. The introduction of respiratory precautions, such as wearing facemasks, could have contributed to decreasing PM permeation and reducing the risk of respiratory diseases^11,12^. Guan et al. showed that real facemasks attenuate pollution-induced effects on airway inflammation^41^. The ability of the respiratory system to remove contaminants from inhaled air depends on the type of filter or absorbent materials, respiratory type, and facial fitting^42^. Certified masks, including N95 and N99, exhibited high performance in particle penetration (filtration efficiencies > 98%) but demonstrated limited effectiveness in the removal of gaseous reactive oxygen species^43^. This observation substantiates our findings, indicating that, unlike PMs, the effects of NO_2_, SO_2_, and O_3_ on the hospitalization rate for respiratory diseases did not exhibit distinctive variations before and after the COVID-19 pandemic.

Secondly, a significant disruption in seasonal respiratory viruses during the COVID-19 pandemic may explain the substantial reduction in hospitalization due to respiratory diseases compared to non-respiratory diseases. Our results are consistent with previous studies indicating a drastic reduction in influenza and other seasonal respiratory viruses during the COVID-19 pandemic^44^. In alignment with existing research^8,9^, PM_10_ and PM_2.5_ concentrations significantly decreased in six Korean cities during the Post-COVID-19 outbreak period. This supports the notion that reduced PM, coupled with mitigation measures, and the competitive capabilities of severe acute respiratory syndrome coronavirus-2, may have contributed to diminishing seasonal respiratory viruses. These viruses are recognized as the primary triggers for acute exacerbations of COPD and asthma during the COVID-19 pandemic.

Despite these insights, it is important to note that our study predominantly focused on meteorological factors as covariates in the analysis of the relationship between air pollution and respiratory diseases. However, an extensive literature review reveals a notable absence of explicit mention or detailed discussion concerning the inclusion of other potential covariates such as socioeconomic factors, population density, or public health interventions^45,46^. This research gap is critical as these elements could significantly affect the study outcomes^47^. The omission of these additional covariates in numerous analyses highlights a potential area for further research. Socioeconomic factors, population density, and public health interventions, acknowledged as influential determinants of health outcomes, may alter the impact of air pollution, underscoring the need for a more comprehensive approach in subsequent studies.

Our study has some limitations. First, we used single-exposure DLNMs. Due to multicollinearity between PM_10_ and PM_2.5_, both variables could not be simultaneously integrated into the regression analysis. Second, as the post-COVID-19 outbreak period lasted only 1 year, Cis were widely estimated in the analysis. The availability of data from 2021 and beyond from the Health Insurance Agency could facilitate more stable CI estimation. Third, evaluating the proper performance of NPIs, including correct facial mask usage, poses challenges. Lastly, factors related to healthcare-seeking behaviors could not be considered. The focus on meteorological factors as covariates leaves room for future studies to explore additional factors, including healthcare system adaptations and population immunity, which could further refine our understanding of the observed patterns.

In conclusion, our study reveals a dynamic shift in the impact of PM_10_ and PM_2.5_ on the risk of admission for respiratory diseases during the COVID-19 outbreak. The observed changes underscore the effectiveness of NPIs, such as the use of facial respirators and adherence to social distancing, in mitigating air pollutant-related respiratory diseases. These in mitigating for local authorities, offering a reference point to formulate protective measures and inform the development of public health policies.

### Supplementary Information

Supplementary Figures.

## Data Availability

The datasets generated during and/or analyzed during the current study are available from the corresponding author on reasonable request. To encourage the easy use of our proposed optimization algorithm, we provided a reproducible tutorial on GitHub^25^. For more detailed instructions on the tutorial, please refer to the webpage.
