# Supplementary material for: Atmosphere particulate matter and respiratory diseases during COVID-19 in Korea
Source: Sci Rep. 2024 May 2;14:10074. doi: 10.1038/s41598-024-59643-x (PMC11066041; doi:10.1038/s41598-024-59643-x)
Supplement: Supplementary file 1 — Supplementary Figures. [file 41598_2024_59643_MOESM1_ESM.pdf]

[Supplementary file]

Atmosphere particulate matter and respiratory diseases during COVID-19 in Korea

Running title: PM and respiratory diseases during COVID-19

Ji Young Hong, MD, PhD [1¶], Taemo Bang, BS-MS [2¶], Sun Bean Kim, MD, PhD [3¶], Minwoo Hong, MA[4], Jaehun Jung, MD, PhD [4,5*],

Affiliations:

[1] Division of Pulmonary, Allergy and Critical Care Medicine, Department of Internal Medicine, Chuncheon Sacred Heart Hospital, Hallym University Medical Center, Chuncheon-si 24253, Gangwon-do, Republic of Korea

[2] AI Product Team, Gmarket, Seoul, Republic of Korea

[3] Department of Internal Medicine, Division of Infectious Diseases, Korea University College of Medicine, Seoul, Republic of Korea

[4] Department of Preventive Medicine, Gachon University College of Medicine, Incheon, Republic of Korea

[5] Artificial Intelligence and Big-Data Convergence Center, Gil Medical Center, Gachon University College of Medicine, Incheon, Republic of Korea

¶ These authors contributed equally to this work as first authors.

***Corresponding author:**

Jaehun Jung, MD, PhD

Department of Preventive Medicine, Gachon University College of Medicine,

38-13, Dokjeom-ro 3beon-gil, Namdong-gu, Incheon, 21565, Republic of Korea.

Tel: +82-10-6359-3201

Fax: +82-32-458-2608

E-mail: eastside1st@gmail.com; ORCID ID: https://orcid.org/0000-0002-4856-3668

Contents

Supplementary Figure 1……………………………………………………………3

Supplementary Figure 2…………………………………………………………...4

Supplementary Figure 3…………………………………………………………...5

Supplementary Figure 4…………………………………………………………...6

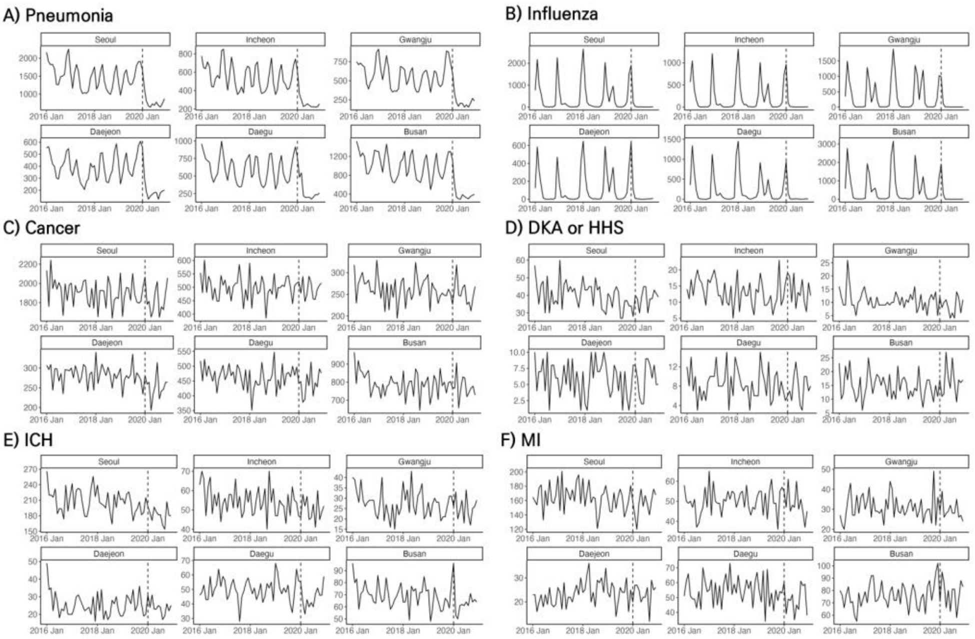

Supplementary Figure 1. (A)-(F) The monthly admissions of other diseases in metropolitan cities, South Korea, from 2016-2020. The area following the dashed line indicates the Post-COVID-19 period. Diabetic ketoacidosis (DKA); Hyperosmolar hyperglycemic state (HHS); Intracranial hemorrhage (ICH); Myocardial infarction (MI).

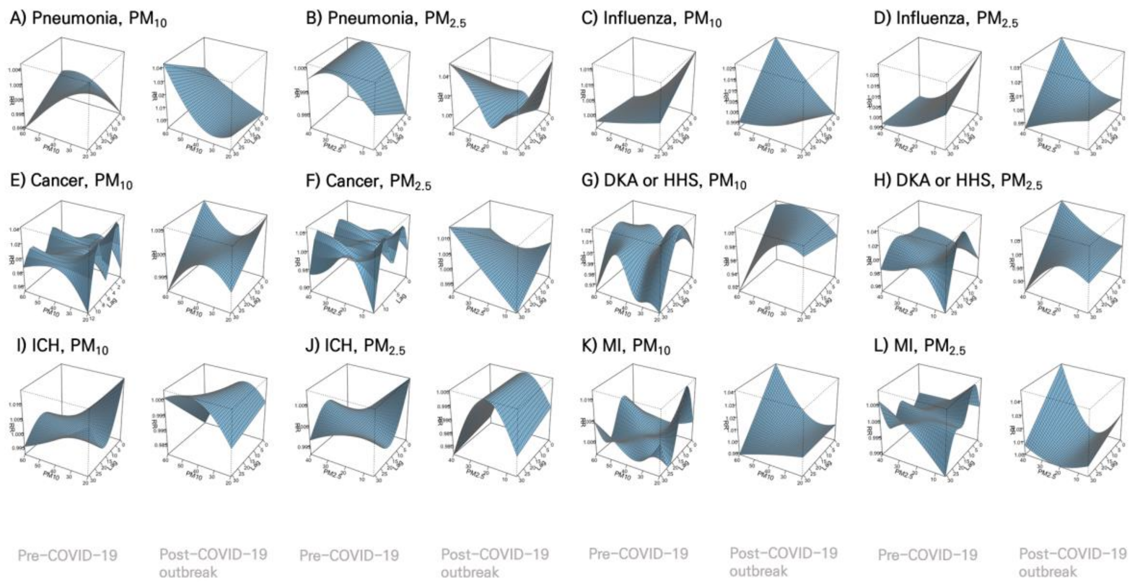

Supplementary Figure 2. Overall PM_{10} and $PM_{2.5}$ effect on admissions of other diseases by 31 lag days in the Pre-COVID-19 and Post-COVID-19 outbreak period as 3D plots for multivariate meta-analyses. A) and B) represent the results for Pneumonia. C) and D) represent the results for Influenza. E) and F) represent the results for Cancer. G) and H) represent the results for Diabetic ketoacidosis (DKA) or Hyperosmolar hyperglycemic state (HHS). I) and J) represent the results for Intracranial hemorrhage (ICH). K) and L) represent the results for Myocardial infarction (MI).

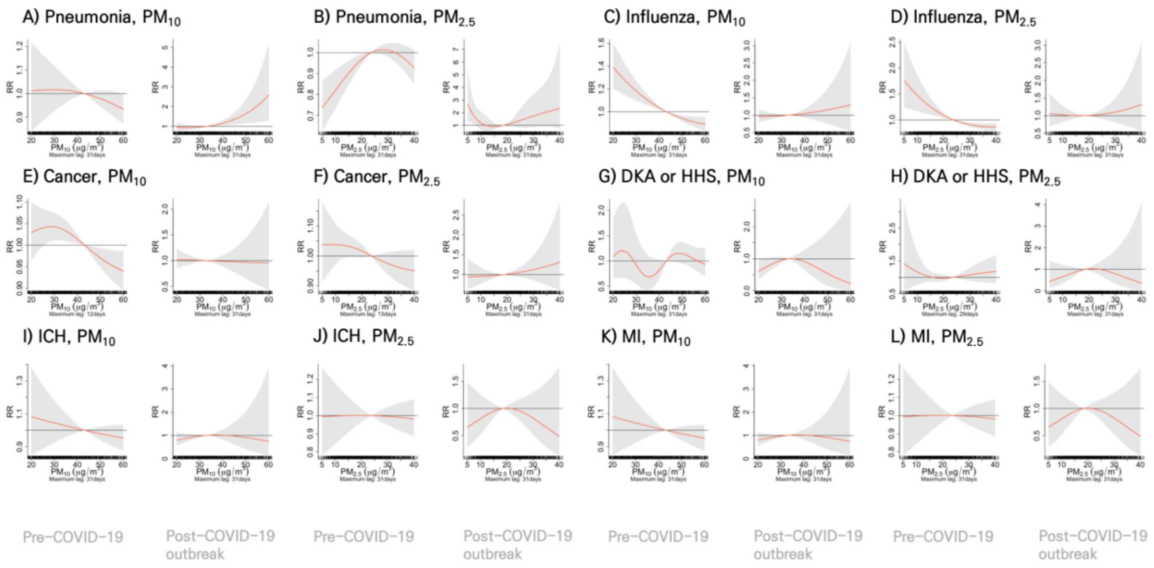

Supplementary Figure 3. Cumulative PM_{10} and $PM_{2.5}$ effect of lag days on admissions of other diseases as overall cumulative association plots for multivariate meta-analyses. A) and B) represent the results for Pneumonia, which are shown in a row in the Pre-COVID19 and Post-COVID19 outbreak period. C) and D) represent the results for Influenza, which are shown in a row in the Pre-COVID19 and Post-COVID19 outbreak period. E) and F) represent the results for Cancer, which are shown in a row in the Pre-COVID19 and Post-COVID19 outbreak period. G) and H) represent the results for Diabetic ketoacidosis (DKA) or Hyperosmolar hyperglycemic state (HHS), which are shown in a row in the Pre-COVID19 and Post-COVID19 outbreak period. I) and J) represent the results for Intracranial hemorrhage (ICH), which are shown in a row in the Pre-COVID19 and Post-COVID19 outbreak period. K) and L) represent the results for Myocardial infarction (MI), which are shown in a row in the Pre-COVID19 and Post-COVID19 outbreak period.

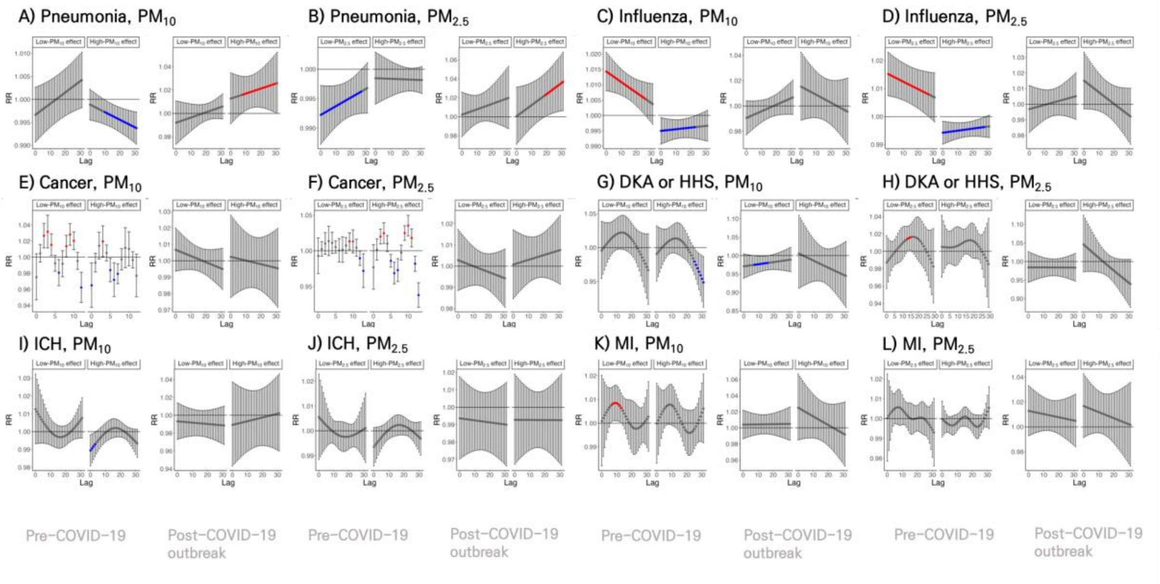

Supplementary Figure 4. Extreme effect of PM_{10} and $PM_{2.5}$ on admissions of other diseases as high-low effect plots for multivariate meta-analyses. High effect and low effect mean a 90th quantile value versus the median value of each PM in Seoul and a 10th quantile value versus the median value of each PM in Seoul respectively. The dot means the point estimator of the RR, and the bar means the 95% interval. If the confidence interval is greater than 1, the dot has a red color, and if it is less than 1, the dot has a blue color. A) and B) represent the results for Pneumonia, which are shown in a row in the Pre-COVID19 and Post-COVID19 outbreak period. C) and D) represent the results for Influenza, which are shown in a row in the Pre-COVID19 and Post-COVID19 outbreak period. E) and F) represent the results for Cancer, which are shown in a row in the Pre-COVID19 and Post-COVID19 outbreak period. G) and H) represent the results for Diabetic ketoacidosis (DKA) or Hyperosmolar hyperglycemic state (HHS), which are shown in a row in the Pre-COVID19 and Post-COVID19 outbreak period. I) and J) represent the results for Intracranial hemorrhage (ICH), which are shown in a row in the Pre-COVID19 and Post-COVID19 outbreak period. K) and L) represent the results for Myocardial infarction (MI), which are shown in a row in the Pre-COVID19 and Post-COVID19 outbreak period.
